# Volatiles Emission by *Crotalaria* *nitens* after Insect Attack

**DOI:** 10.3390/molecules26226941

**Published:** 2021-11-17

**Authors:** Fausto Prada, Elena E. Stashenko, Jairo René Martínez

**Affiliations:** 1 Center for Chromatography and Mass Spectrometry (CROM-MASS), Universidad Industrial de Santander, Bucaramanga 680002, Colombia; fausto.prada@correo.uis.edu.co (F.P.); jmartine@uis.edu.co (J.R.M.); 2 Colombia Research Center for Biomolecules (CIBIMOL), Universidad Industrial de Santander, Bucaramanga 680002, Colombia

**Keywords:** aldoximes, *Crotalaria nitens*, herbivore-induced plant volatiles, purge &amp, trap, solid-phase microextraction, *Utetheisa ornatrix*

## Abstract

Plants are known to increase the emission of volatile organic compounds upon the damage of phytophagous insects. However, very little is known about the composition and temporal dynamics of volatiles released by wild plants of the genus *Crotalaria* (Fabaceae) attacked with the specialist lepidopteran caterpillar *Utetheisa ornatrix* (Linnaeus) (Erebidae). In this work, the herbivore-induced plant volatiles (HIPV) emitted by *Crotalaria nitens* Kunth plants were isolated with solid phase micro-extraction and the conventional purge and trap technique, and their identification was carried out by GC/MS. The poly-dimethylsiloxane/divinylbenzene fiber showed higher affinity for the extraction of apolar compounds (e.g., *trans*-β-caryophyllene) compared to the Porapak™-Q adsorbent from the purge & trap method that extracted more polar compounds (e.g., *trans*-nerolidol and indole). The compounds emitted by *C. nitens* were mainly green leaf volatile substances, terpenoids, aromatics, and aldoximes (isobutyraldoxime and 2-methylbutyraldoxime), whose maximum emission was six hours after the attack. The attack by caterpillars significantly increased the volatile compounds emission in the *C. nitens* leaves compared to those subjected to mechanical damage. This result indicated that the *U. ornatrix* caterpillar is responsible for generating a specific response in *C. nitens* plants. It was demonstrated that HIPVs repelled conspecific moths from attacked plants and favored oviposition in those without damage. The results showed the importance of volatiles in plant–insect interactions, as well as the choice of appropriate extraction and analytical methods for their study.

## 1. Introduction

The coevolutionary process has allowed plants to biosynthesize a great diversity of secondary metabolites that reduce the attack of herbivores [1,2]. Alkaloids, phenylpropanoids, and digestion inhibitors directly repel phytophagous insects [3,4], while herbivory-induced plant volatiles (HIPV) play an important role in the indirect defense of plants, attracting antagonistic natural enemies of herbivores [5,6]. Interactions at different trophic levels are becoming relevant as a phenomenon that shapes the distribution and abundance of plants and insects in natural ecosystems [7,8,9].

Plants of the genus *Crotalaria* (Fam. Fabaceae) or rattlepods, grow as weeds in plains and roadsides of the inter-Andean valleys [10], they produce pyrrolizidine alkaloids as a direct defense against generalist herbivores [11]. In 1982, Thomas Eisner reported for the first time that pyrrolizidine alkaloids from *Crotalaria pallida* were related to the chemical defense of the moth *Utetheisa ornatrix* (Linnaeus) (Lepidoptera: Erebidae) [12]. The insect can sequester, transform, and use these alkaloids without negative effects on the fitness of *U*. *ornatrix* [13,14,15]. Many specialist herbivores have physiological adaptations that allow them to tolerate toxins [16,17]. Previous reports [18,19] showed that, under laboratory experiments, *U. ornatrix* caterpillars sequestered and accumulated more pyrrolizidine alkaloids from *Crotalaria nitens* Kunth than other species evaluated. However, the attack of the caterpillar had no effect on the concentration of these alkaloids in the leaves of *C. nitens* [19]. This result led to the hypothesis that volatiles may be involved in the interaction of *C. nitens* with *U. ornatrix*, and to our knowledge this appearance has not been evaluated in any species of the genus *Crotalaria*.

Knowledge about volatiles in plants, their regulation, biosynthesis, and ecological importance in ecosystems has increased considerably since they were first reported around thirty years ago [8,9,20], especially due to their potential use as biological control tools in agricultural systems [21] by manipulating the behavior of insect pests or their natural enemies [22,23,24]. Therefore, it requires the development of sensitive extraction and analytical methods for their identification and analysis. The most common sampling method is the purge and trap (P&T) of the volatiles in ad/absorbent materials [25,26]. However, solid-phase microextraction (SPME), developed by Pawliszyn et al. [27] in the early ‘90s, has proven to be a valuable tool for the extraction of volatile compounds due to its sensitivity and simplicity of handling without the use of solvents [28]. The technique has recently been applied to the detection of HIPV in plant–insect interaction experiments [29]. Here, we compared the two methods—P&T and SPME—for the extraction and identification of HIPV emitted by *C. nitens* leaves.

HIPVs can generate contrasting effects, repelling, or attracting conspecific adult herbivores. For instance, the lepidopteran moths *Heliothis virescens* (Fabricius) (Noctuidae) [30], *Trichoplusia ni* (Hübner) (Noctuidae) [31], and *Manduca sexta* (Linnaeus) (Sphingidae) [32] are repelled by HIPVs released by the attacks of their caterpillars, while other conspecific adult species are attracted to infested plants, such as the lepidoptera *Epiphyas postvittana* (Walker) (Tortricidae) [33], *Busseola fusca* (Fuller) (Noctuidae), *Sesamia calamistis* (Hampson) (Noctuidae), and *Chilo partellus* (C. Swinhoe) (Crambidae) [34]. However, it is unknown whether the moths of the specialist insect *U*. *ornatrix* are attracted to or repelled by the blend of HIPVs emitted by *C. nitens*.

The objective of this work was to determine the composition and temporal dynamics of HIPVs release in *C. nitens* leaves attacked by *U*. *ornatrix* caterpillars or injured by mechanical means. Sampling was performed by trapping the volatiles in the Porapak™-Q adsorbent in a conventional P&T system and on a poly-dimethylsiloxane/ divinylbenzene (PDMS/DVB) fiber supported in an SPME system. The identification of the volatiles was carried out by GC/MS. We studied whether the HIPVs blend emitted by *C. nitens* could modify the oviposition behavior of conspecific adult moths. The present study contains the characterization of HIPVs emitted by *C. nitens* plants and demonstrates their importance in the interaction with the specialist moth *U. ornatrix*.

## 2. Results

### 2.1. GC/MS Analysis of Volatile Compounds Isolated by SPME and P&T

**Figure 1:**
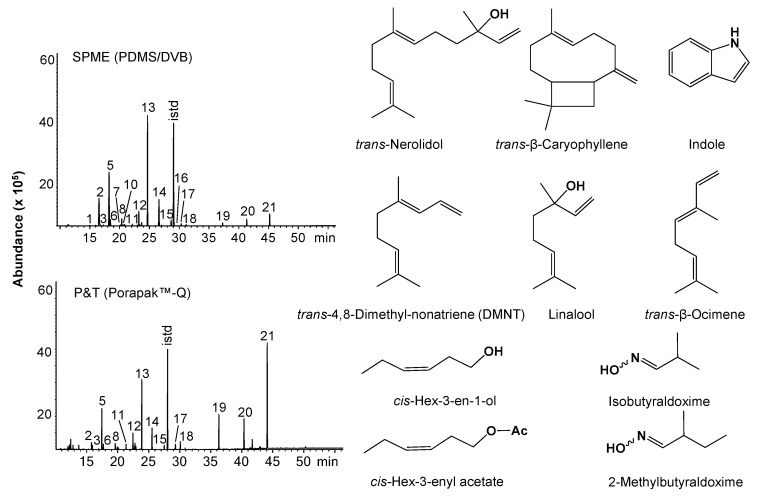
Chromatographic profiles of HIPVs emitted by *C*. *nitens* leaves and the chemical structure of the main volatile compounds. SPME (PDMS/DVB, 65 µm) and P&T (Porapak™-Q) sampling. GC/MS analysis (DB-WAX column, 60 m). Table 1 contains peak identification; istd, methyl undecanoate.

Many plant species emit volatiles in response to biotic and abiotic factors. The volatiles emitted by *C. nitens* leaves attacked by *U. ornatrix* caterpillars were sampled by two methods, one with a PDMS/DVB fiber of 65 µm (SPME) and the other using the P&T system with 25 mg of Porapak™ adsorbent. The GC/MS analysis of the volatiles allowed the identification of 21 compounds ( >0.05%) (Table 1 and Figure 1) belonging to the families of C_6_-fatty acid derivatives called green leaf volatiles (GLV), terpenoids, aromatic, and aldoximes.

**Figure 2:**
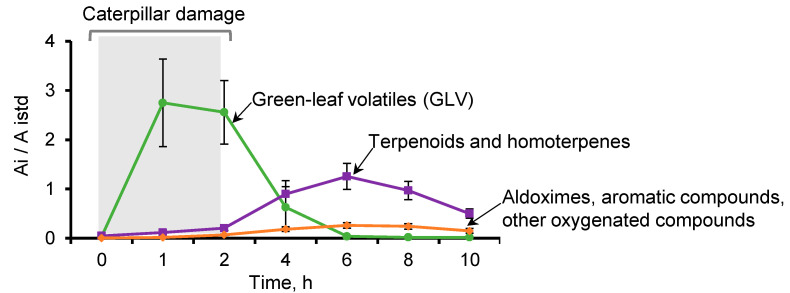
HIPV emission by *C*. *nitens* leaves attacked by *U*. *ornatrix* caterpillars. The caterpillars were fed for the first two hours, then removed from the plant. Volatiles were extracted using SPME. The ratio between the sum of areas of the group of compounds of interest A_i_ and the area of the internal standard A_istd_ (methyl undecanoate) is reported. Values are the mean ± standard error (*n* = 7).

The temporal release dynamics of HIPVs in *C. nitens* leaves was determined every hour during the first two hours of insect attack and every two hours, up to 10 h, after removing the caterpillars (Figure 2). At the start of the attack, GLVs (*cis*-hex-3-enal, hexyl acetate, *cis*-hex-3-enyl acetate, and *cis*-hex-3-en-1-ol) and the homoterpene *trans*-4,8-dimethyl-nonatriene (DMNT) were mainly detected [35]. During this time, the SPME fiber extracted 86.9% of GLVs and 8.9% of terpenes, while 97.6% of the compounds were GLVs and only 1.4% were terpenoids, using the P&T system. The SPME fiber isolated some terpenoids, such as, *trans*-β-ocimene (2.9%), linalool (0.4%), α-humulene (0.09%), and germacrene D (0.15%), not registered in the extract of the P&T system.

After two hours of attack, the release of GLVs was reduced to 1.9% and 1.2% measured by SPME and P&T, respectively; however, the emission of terpenoids, aromatics, aldoximes, and other oxygenated compounds increased reaching a maximum at six hours. At this time, the proportion of terpenoids (mainly *trans*-β-ocimene, linalool, *trans*-β-caryophyllene, α-humulene, *trans*, *trans*-α-farnesene, and *trans*-nerolidol), including the homoterpene DMNT, raised their concentration to 77.2% and 63.7% extracted by SPME and P&T methods, respectively. The aromatic compounds (methyl salicylate, methyl anthranilate and indole) were detected at 2.7% (SPME) and 22.2% (P&T). The aldoximes isobutyraldoxime and 2-methylbutyraldoxime were extracted by the two methods at 11.5% (SPME) and 8.7% (P&T). Such relatively simple oxime structures have been reported in approximately 22 different plant species [41]. Their identification was confirmed by mass spectra analysis and comparison of experimental data with those from the database (NIST, 2017) as shown in Figures S1 and S2. At six hours after the attack, the SPME fiber extracted other compounds, e.g., *allo*-ocimene (0.18%), 3-octanol (0.14%), and germacrene D (0.6%), that were not detected with the P&T method.

The PDMS/DVB fiber adsorbed a higher proportion of nonpolar compounds (e.g., *trans*-β-ocimene, DMNT and *trans*-β-caryophyllene), while the adsorbent Porapak™-Q extracted a higher proportion of more polar compounds (e.g., *trans*-nerolidol, methyl salicylate, methyl anthranilate, and indole) (Table 1).

### 2.2. Volatile Compounds from C. nitens after Either Mechanical Damage or Caterpillar Attack

**Figure 3:**
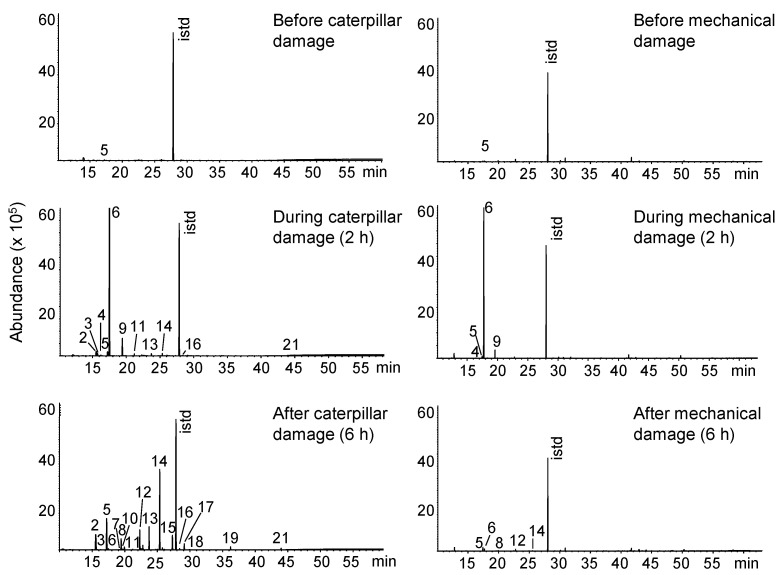
Volatile compounds released by *C*. *nitens* leaves after attack by *U*. *ornatrix* caterpillars or after mechanical damage. Peak identification appears in Table 1; istd, methyl undecanoate.

To determine if the volatiles emitted by *C. nitens* leaves was a specific response to the attack of the caterpillar, the compounds released after mechanically damaging the leaves with a scalpel were measured and compared with those compounds emitted after insect attack. The areas of damage done by both treatments were similar. Plants without attack only emit small amounts of DMNT (Figure 3). Soon after the mechanical damage, the leaves of *C. nitens* emitted the same GLVs (*cis*-hex-3-enyl acetate and *cis*-hex-3-en-1-ol) that they emitted after the insect attack. However, after two hours, the *C. nitens* leaves did not emit aromatic compounds and the emission of terpenoids and aldoximes decreased drastically compared to the volatile compounds emission made by the leaves after being damaged by the caterpillars.

### 2.3. Conspecific Moths Repelled by Volatiles Induced by Herbivory with U. ornatrix Caterpillars

**Figure 4:**
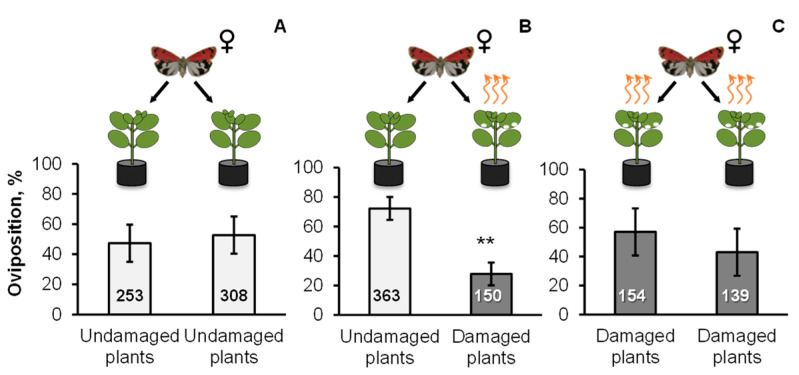
Oviposition preference of *U*. *ornatrix* female moths on *C*. *nitens* leaves. (**A**) On both sides of one carboard box were placed one undamaged plant. (**B**) On one side of a second box an undamaged plant was used and, on the other side, a plant that had been attacked by six third-instar caterpillars was included. (**C**) A third box contained two damaged plants. Welch’s two sample *t*-test (** *p* < 0.01). Values are the mean ± standard error (*n* = 7). Each plant pair was used only once. Numbers included in bars correspond to the total number of eggs laid by moths across all replicates.

Our results showed that *C. nitens* leaves emitted various GLVs, terpenoids, homoterpenes, aldoximes, and aromatic compounds after caterpillar attack, but not after mechanical damage. It has been reported that HIPVs can modify the herbivorous insect behavior [2]. The last experiment showed that HIPVs emitted by *C. nitens* leaves, after the attack by the *U. ornatrix* caterpillars, repelled the conspecific female moths (*df* = 12, t = −4.05, *p* = 0.0016, Figure 4). The moths preferred to lay their eggs on undamaged plants rather than on those attacked by the caterpillars. During the 24 h of measurement, the five moths laid an average of 52 and 21 eggs on each undamaged and damaged plant, respectively. The moths from the control box having both undamaged plants showed in average 40 eggs/plant compared to the 21 eggs/plant in the control box with the two damaged plants.

## 3. Discussion

The secondary metabolites produced by plants are important for their survival in all ecosystems, for adaptation to environmental changes and for ecological interactions with other organisms [4]. However, the identification and release dynamics of the volatiles emitted in *Crotalaria* genus plants in response to attack by herbivorous insects are still unknown [9]. In the present investigation, it was shown that *C. nitens* plants recognized the wound of the specialist caterpillar *U. ornatrix* and emitted HIPVs in response to its attack. To our knowledge, it is the first time that HIPVs are identified for a species of *Crotalaria*. The volatiles emitted by the leaves of *C. nitens* modified the behavior of *U. ornatrix* moths, significantly reducing their oviposition on previously attacked plants and preferring undamaged plants.

The volatiles released by plants play an important role in interactions with other organisms [2], therefore sensitive extraction methods are needed that accurately represent the mixture of compounds released [25,42]. In this work, we studied the volatiles emitted by *C. nitens* leaves by sampling with SPME and with a conventional P&T system. Twenty-one volatile compounds released by the *C. nitens* leaves were identified, belonging to the GLVs, terpenoid, homoterpene, aldoxime, and aromatic compound families. The sampling with the PDMS/DVB fiber was more sensitive and managed to isolate some terpene compounds that could not be detected in the extracts obtained by the P&T extraction. The reason is that the SPME fiber allowed the analytes to be concentrated on the fiber and then desorbed directly into the injection port of the chromatographic system, whereas with the P&T method, the compounds trapped in the sorbent were diluted with the extracting solvent before their analysis. The volatile compounds extraction using SPME was simpler to operate and did not require the use of solvents and therefore eliminated possible interferences and cross-contamination compared to the P&T method. However, during the collection of HIPVs by SPME, the container accumulates moisture, and this condition can interfere with the physiological processes of the plant or the insect [25]. This problem did not occur with the P&T method, due to the injection of filtered air into the system from the outside. However, the constant air flow increased the adsorption of pollutants that could not be retained by the activated carbon filters installed at the inlet of the system.

The *C. nitens* leaves emitted a wide variety of volatiles in response to the attack by the specialist caterpillar *U. ornatrix*. GLVs were the first compounds emitted by *C. nitens* leaves [35,43]. Subsequently, the terpenoids, the homoterpene DMNT, aldoximes, and aromatic compounds were released with a maximum emission at six hours after the attack. When the *C. nitens* leaves were subjected to mechanical damage, large amounts of GLVs were detected, but after two hours, the volatile emissions decreased dramatically compared to those generated upon insect attack. This result indicates that the *U. ornatrix* caterpillars were responsible for generating a specific response in *C. nitens* leaves. The ability of plants to distinguish types of damage, allows them to reduce the expenditure of valuable resources for defense in situations where it is not necessary. Some plants recognize the attack of the caterpillar by the presence of molecules such as volicitin or its derivatives, present in the oral secretion of *Spodoptera exigua* (Hübner) (Lepidoptera: Noctuidaese), which induce the emission of HIPVs from corn [44] or cotton [45] leaves. The results of the present study suggest that *C. nitens* plants distinguished the attack of the *U. ornatrix* caterpillars and activated a series of biochemical reactions that promoted volatile compounds release [1]. However, further research is required to determine whether the HIPVs induction was generated by specific molecules, enzymes, or microorganisms from the caterpillars’ saliva.

The volatiles released by *C. nitens* leaves had been reported as herbivory-induced volatiles in other plants [46]. HIPVs are detected by insects to locate/avoid plants to use as oviposition sites [33,34,47]. In the present work, the blend of the HIPVs emitted by *C*. *nitens* leaves after the caterpillar wounding significantly reduced the oviposition of the conspecific moths in damaged plants (28%) preferring to lay the eggs on those undamaged (72%). These results were akin to those reported in other species of Lepidoptera. For instance, De Moraes et al. [30] observed that *H*. *virescens* moths selected non-infested tobacco plants (*Nicotiana tabacum*) to lay the eggs (80%) and avoided the infested plants. The same behavior was registered for *T. ni* [31], *M. sexta* [32], *Manduca quinquemaculata* (Haworth) (Lepidoptera: Sphingidae) [2], or *Spodoptera frugiperda* (Smith) (Lepidoptera: Noctuidae) [48]. Changes in either the presence or relative abundance of the volatile compounds emitted by the plant upon caterpillar damage could have a completely different meaning for the moth, causing the plant not to be recognized as host [47]. Indeed, herbivore-induced volatiles of *C. nitens* included linalool (11–13%), an oxygenated monoterpene known to have oviposition-deterrent properties against many insect species [2,49,50]. *trans*-β-Ocimene and DMNT, emitted by *C. nitens* leaves in relative amounts of 9–19%, have shown to be repellents of stemborer moths, including *Busseola fusca* (Füller) (Lepidoptera: Noctuidae), and attractants of pest’s natural enemies in the push–pull system in maize crops [23], and DMNT also deterred moths of the leaf worm, *Spodoptera littoralis* (Boisd.) (Lepidoptera: Noctuidae) from damaged plants of cotton (*Gossypium hirsutum* L.). The ability of moths to recognize changes in plant volatiles could have the benefit of avoiding host plants already infested with insects, which reduces the competition for food resources of their future offspring [30,47].

Other HIPVs identified in the present work have shown to be involved in the interaction between different organisms. GLVs, which first appeared due to herbivory or mechanical damage, have been reported in damaged leaves of maize seedlings [5,35], *A. thaliana* [51], cotton [52], and black poplar (*Populus nigra*) [53], among others. GLVs have ecological functions such as attracting or repelling insects [54], *cis*-hex-3-enyl acetate showed to increased herbivory defenses in maize [55] and activated defense genes in black poplar [56]. *trans*-β-Caryophyllene has been reported as repellent of *Diaphorina citri*, a bacteria-spreading insect in citrus crops [57], and to induce resistance against microbial pathogens in neighboring plants [58]. Linalool, *trans*-β-ocimene, DMNT, and methyl salicylate attracted female mite (*Phtoseiulus persimilis*), a natural predator of another mite (*Tetranychus urticae*), considered a pest in lima bean plants (*Phaseolus lunatus*) [59]. Although *C. nitens* emitted few amounts of *allo*-ocimene (0.18%), this compound has shown antifungal properties in *A. thaliana* [60]. α-Humulene reduced the mating ability of *Ceratitis capitata* females and males, a pest of various crops in the Mediterranean [61], and the *trans*-nerolidol was tested to induce resistance against herbivores and pathogens in tea plants [62].

The two most abundant HIPV families in *C. nitens* leaves were GLVs and terpenoids. However, *C. nitens* HIPVs also contained two aldoximes (isobutyraldoxime and 2-methylbutyraldoxime) in relative amounts of 9–11%. Aldoximes are imine-type compounds derived from amino acids that have been reported as end products or intermediates to be further converted to nitriles and other metabolites [41]. Isobutyraldoxime and 2-methylbutyraldoxime are herbivore-induced compounds reported in *Phaseolus vulgaris* [41] and species of the genus *Populus* (Salicaceae) [63], among others. They are precursors of volatile nitriles in *P. trichocarpa* with repellent activity against the caterpillars *Lymantria dispar* (Lepidoptera: Erebidae) [64]. The 2- and 3-methylbutyraldoximes attracted the wasp *Glyptapantheles liparidis*, a parasitoid of the caterpillars *L. dispar* in *P. nigra* trees [65]. Regarding aromatic compounds, the leaves of *C. nitens* emitted methyl salicylate (0.3–1.6%), methyl anthranilate (0.3–3%), and indole (2–18%). Methyl salicylate enhanced the growth of *Lecanicillium lecanii*, an entomopathogenic fungus, natural enemy of the aphid *Lipaphis erysimi* [66]. Methyl anthranilate, a naturally occurring volatile found in strawberries, reduced fungal growth that negatively affects fruit shelf life [67], and indole has been involved in plant–plant communication. It primmed the emission of volatiles (GLV and terpenoids) within the same plant and in neighboring plants that prepared them for future attacks by *S. littoralis* caterpillars [35].

Every HIPV compound emitted by *C. nitens* leaves has been reported to influence the ecological interactions of plants with other organisms. They can directly repel herbivores, serve as important foraging cues for natural enemies of the phytophagous insect, demonstrate antifungal activity, or prime non-attacked plant tissues and neighboring plants to respond more strongly to future attacks. Determining the single compound or blend of compounds emitted by *C. nitens* and associated with ecological functions might be a promising approach in this context. In this regard, the development and application of efficient methods for the extraction of target compounds, together with highly sensitive analytical instrumentation, become indispensable tools for a deep understanding of the ecological role of volatiles and their relevance in plant-insect interactions and the design of volatile-based agricultural strategies.

## 4. Materials and Methods

### 4.1. Reagents

*cis*-Hex-3-enal solution (>50%), the mixture of isomers *cis*-, and *trans*-β-ocimene (>90%), *cis*-hex-3-enyl acetate (>98%), isobutyraldoxime (>80%), *cis*-hex-3-en-1-ol (>98%), oct-1-en-3-ol (>98%), linalool (>95%), *trans*-β-caryophyllene (>98%), α-humulene (>96%), *trans*-nerolidol (>90%), indole (>99%), and methyl undecanoate (>99%) were obtained from Sigma-Aldrich (St. Louis, MO, USA or Steinheim, Germany). Methanol and dichloromethane were HPLC-grade from Merck (Darmstadt, Germany). The Porapak™-Q 80/100 mesh adsorbent was from Supelco (Bellefonte, PA, USA). The C_6_-C_25_ *n*-alkanes mixture was purchased from AccuStandard, Inc. (New Haven, CT, USA). Helium (99.995%) for GC analysis was acquired from Messer (Bucaramanga, Colombia).

### 4.2. C. nitens Plants

The seeds were collected from a natural population in the Andean mountains of the department of Santander, Colombia (6°56′41.5″ N, 73°02′05.8″ W, 1144 m above mean sea level), in June 2015. Botanical identification of *C. nitens* (voucher COL579431) was carried out at the National Herbarium of the Institute of Sciences at the National University of Colombia, Bogotá. A population of *C. nitens* plants was established in experimental plots of the CENIVAM research center. The *C. nitens* plants for the experiments were propagated in sand beds with daily irrigation and under natural conditions, after 15 days they were individually transplanted into pots (1 L) containing soil and rice chaff in a 7:3 ratio, respectively. The seedlings grew under greenhouse conditions with a relative humidity of 60 ± 10%, a temperature of 24 ± 3 °C and a 12 h photoperiod. They were watered every two days and fertilized weekly with a solution (~50 mL, 1 g/L) of water-soluble fertilizer N-P-K-MgO: 11-27-12-2. Ten- to twelve-week-old plants were used for the experiments.

### 4.3. U. ornatrix Caterpillars and Moths

Approximately thirty caterpillars were collected from a natural population of *Crotalaria* spp. plants in Santander, Colombia (6°59′49.1″ N, 73°22′11.4″ W, 299 m above mean sea level) in September 2018. The caterpillars were placed in a plastic container (32 cm × 28 cm, 16 cm high) covered in tulle fabric. They were placed in an open room with air circulation, protected from rain and at room temperature (18–26 °C). The caterpillars were fed daily ad libitum with leaves or seeds of *C. nitens* from the culture previously established in the experimental plots. The pupae were carefully placed in butterfly houses constructed of cardboard boxes (41 cm × 41 cm, 60 cm high) covered with tulle fabric. When they emerged, the moths were fed twice a day with an aqueous solution of honey (5%), according to the methodology of Martins et al. [68]. One-month-old *C. nitens* plants were placed inside the butterfly house for the moths to lay their eggs under their leaves. Eggs were collected daily and used to continue rearing *U. ornatrix* or to generate caterpillars and moths for experiments. The taxonomic identification showed a 99.9% similarity with *U. ornatrix* determined by COI gene sequencing [69]. The life cycle of *U. ornatrix* was similar to that described by Signoretti et al. [70]. The third or fourth instar caterpillars were brought to the laboratory 24 h before the experiments to allow their conditioning.

### 4.4. Volatile Compound Emission by C. nitens Leaves

To establish the variation in the emission of volatiles that occur in *C. nitens* leaves before, during and after the *U. ornatrix* caterpillars attack or after mechanical damage with a scalpel, *C. nitens* plants were randomly placed in setups for three treatments. Plants of the first group were subjected to the attack of *U. ornatrix* caterpillars (6/plant) for two hours [5]. Leaves N° 4 and N° 5 of the second group of plants were scraped on the surface with the scalpel (mechanical damage) similar to the area generated by the damage by the caterpillars [5,30]. Plants of the third group were used as a reference (control). Plants were transferred to the laboratory one day before the experiments. The volatiles emitted by *C. nitens* leaves were sampled by SPME and P&T methods and analyzed by GC/MS. 

#### 4.4.1. SPME Isolation of *C. nitens* Volatile Compounds 

The extraction of volatiles by SPME was carried out following the methodology described by Stashenko et al. [28], with modifications. One branch of each *C. nitens* plant (*n* = 7) with about ten leaves was placed inside a container (10 cm × 10 cm × 10 cm) made of acrylic material. The stem of the branch was protected with cotton so as not to hurt the plant when closing the system. The container had an attachment for inserting the SPME fiber holder (PDMS/DVB coated, 65 µm, Supelco, Bellefonte, PA, USA). The fiber was previously exposed (5 s, 24 °C), to the headspace of a flask (2 mL) containing methyl undecanoate (30 mg, internal standard), and then placed for 60 min in the headspace of the container. The volatiles were sampled at 1, 2, 4, 6, 8, and 10 h from the start of the experiment (around 9:00 a.m.). Compounds trapped in the fiber were desorbed for 10 min at the injection port of a GC/MS (DB-WAX column, 60 m). The acrylic container was flushed with methanol prior to each experiment and purged with nitrogen gas between measurements. Fiber, container, and plant blanks were performed prior to caterpillar attack or mechanical damage.

#### 4.4.2. P&T Isolation of *C. nitens* Volatile Compounds 

The volatiles emitted by *C. nitens* leaves were measured in a multiple air supply system, as described by Erb et al. [35]. The assembly consisted of borosilicate glass bottles 50 cm high × 17 cm in diameter. The pots with the *C. nitens* plants (*n* = 7) were wrapped in aluminum foil. The plants were individually placed in each glass jar and hermetically sealed with a ground joint that was fastened on the outside with steel rings and screws. The air, previously purified with activated carbon filters, entered the containers at a speed of 1 L/min and dragged the volatile compounds into the Porapak™-Q trap, at a speed of 0.8 L/min. The volatiles traps consisted of 7 cm glass tubes with 25 mg of the adsorbent, which was held in place by a fine metal mesh on one side and some fiberglass supported by a piece of Teflon (2 mm long) in the other one. The traps were placed horizontally in one of the outlets at the top of each container. A hose (6 mm diameter) was connected to the volatiles trap and then to a vacuum pump through a flow regulator. The volatiles were sampled at hours 1, 2, 4, 6, 8, and 10 from the start of the experiment (around 9:00 a.m.). Before each test, the Porapak™-Q from each trap was flushed with dichloromethane (3 mL) and blank samples of the container and plant were taken prior to caterpillar attack or mechanical damage. Immediately after removing the trap from the system, the compounds were eluted with dichloromethane (150 µL) containing methyl undecanoate as an internal standard (9.8 ng/µL). The dichloromethane extract with the analytes was collected in glass inserts (0.2 mL, Supelco, Bellefonte, PA, USA) inside a chromatography vial and 2 μL was injected into the GC/MS (DB-WAX column, 60 m).

### 4.5. GC/MS Analysis and Identification of C. nitens Volatile Compounds

The identification of volatile compounds was based on chromatographic (t_R_ and LRI) and spectrometric (comparison of experimental mass spectra with those of databases and reference materials) criteria. A mixture of C_6_-C_25_ *n*-alkanes was used for the determination of LRI, which followed the equation proposed by van Den Dool and Kratz [71]. Chromatographic data were obtained on DB-5MS ((J&W Scientific, Folson, CA, USA) capillary columns of 60 m × 0.25 mm id, coated with 5% -phenyl-PDMS (0.25 μm, df)) and on a DB-WAX ((J&W Scientific, Folson, CA, USA) of 60 mx 0.25 mm id, coated with PEG (0.25 μm, df) following the procedure described previously [28]. The experimental values of the LRI were compared with those reported in the literature [36,37,38,39,40]. In all experiments, the chromatographic areas of the detected volatiles were used to calculate the relative abundances (relative GC area, %) or the amount relative to that of the internal standard (A_i_/A_istd_).

### 4.6. U. ornatrix Oviposition on C. nitens

It was evaluated whether the total mixture of HIPVs emitted by *C. nitens*, after the leaves were attacked by the caterpillar, can modify the behavior of *U. ornatrix* female moths. For this, their oviposition preference was determined on undamaged or caterpillar-attacked *C. nitens* plants. Two *C. nitens* plants were placed in a cardboard box (60 cm × 60 cm × 60 cm) covered with tulle fabric. On one side of the box, a plant without damage was placed, and on the other side, a plant that had been attacked for 2 h by six third-instar *U. ornatrix* caterpillars was included. The caterpillars were removed from the plant before moths were introduced. Five mated six-day-old female *U. ornatrix* moths were released into the box (around 11:00 a.m.). Two additional boxes were used as controls, testing the same oviposition preference; the first box contained two undamaged plants in opposite sides, and the second box had two caterpillar-damaged plants, obtained as described before. The three boxes were placed randomly and separated more than 5 m to avoid the effect of volatiles from the neighboring boxes. The moths were removed after 24 h and the eggs were counted on each plant. The experiment, including the three previously described boxes, was performed on different days (*n* = 7), depending on the availability of moths.

## 5. Conclusions

In this study, it was demonstrated that *C. nitens* plants recognized the attack of the caterpillar *U. ornatrix* by emitting HIPVs such as terpenoids, homoterpenes, aldoximes, and aromatic compounds that were not detected when their leaves suffered mechanical damage. GLVs were the first compounds to appear after insect wounding or mechanical damage. HIPVs modified the behavior of female *U. ornatrix* moths, reducing their oviposition on plants attacked by the caterpillars. Most of the HIPVs emitted by *C. nitens* leaves were reported to have ecological functions in other models of plant-insect interaction and even in plant–plant communication. The SPME sampling method was more sensitive and isolated some terpenes that were not detected by the conventional P&T method. The present investigation contributed to the GC/MS chemical characterization of the compounds associated with the interaction between a *Crotalaria* species and its specialist herbivore *U. ornatrix* and demonstrated the capacity of volatile blends to modify the behavior of insects. More research is required to understand the intricate chemical and ecological interactions between plants, herbivores, and other organisms, which can be harnessed as tools for the protection of more sustainable and environmentally friendly agricultural systems.

## Figures and Tables

**Table 1:** Identification of volatile organic compounds emitted during and after herbivory of *C*. *nitens* leaves.

Peak N° Figure 1	Compound	DB-WAX		DB-5MS		Relative GC Peak Area (%, Mean ± SE, n = 7)			
		LRI Experim.	LRI Reported	LRI Experim.	LRI Reported	During the Attack (2 h)		After the Attack (6 h)	
						SPME	P&T	SPME	P&T
1	cis-Hex-3-enal ^a^	1140	1139 [36]	800	799 [36]	0.2 ± 0.10	10.1 ± 0.78	n.d.	n.d.
2	trans-β-Ocimene ^a^	1250	1250 [36]	1048	1048 [36]	2.9 ± 0.76	n.d.	14 ± 4.2	9 ± 3.1
3	3-Octanone ^b^	1253	1255 [36]	986	985 [36]	1.8 ± 0.45	0.7 ± 0.21	1.1 ± 0.32	1.7 ± 0.44
4	Hexyl acetate ^b^	1269	1264 [37]	1012	1010 [36]	0.5 ± 0.17	0.31 ± 0.09	n.d.	n.d.
5	trans-4,8-Dimethyl-nonatriene (DMNT) ^b^	1306	1302 [38]	1115	1119 [39]	5 ± 1.4	1.2 ± 0.31	19 ± 2.1	8.9 ± 0.93
6	cis-Hex-3-enyl acetate ^a^	1316	1308 [37]	1004	1004 [36]	80 ± 3.8	79 ± 1.7	1.9 ± 0.54	1.2 ± 0.30
7	allo-Ocimene ^b^	1367	1366 [36]	1129	1130 [36]	n.d.	n.d.	0.18 ± 0.08	n.d.
8	Isobutyraldoxime ^a^	1381	–	752	–	n.d.	n.d.	5 ± 1.6	3.4 ± 0.73
9	cis-Hex-3-en-1-ol ^a^	1384	1378 [36]	855	857 [36]	6 ± 1.7	8 ± 1.3	n.d.	n.d.
10	3-Octanol ^b^	1386	1392 [36]	998	993 [36]	n.d.	n.d.	0.14 ± 0.09	n.d.
11	Oct-1-en-3-ol ^a^	1448	1442 [36]	982	980 [36]	1.2 ± 0.35	n.d.	0.7 ± 0.23	0.3 ± 0.25
12	2-Methylbutyraldoxime ^b^	1479	–	856	–	n.d.	n.d.	6 ± 1.9	5.3 ± 0.73
13	Linalool ^a^	1543	1543 [36]	1101	1099 [36]	0.4 ± 0.10	n.d.	13 ± 3.4	11 ± 1.4
14	trans-β-Caryophyllene ^a^	1610	1598 [36]	1422	1420 [36]	0.7 ± 0.17	0.12 ± 0.07	20 ± 3.6	13 ± 2.4
15	α-Humulene ^a^	1683	1667 [36]	1471	1453 [36]	0.09 ± 0.04	n.d.	4.1 ± 0.77	3.1 ± 0.55
16	Germacrene D ^b^	1720	1708 [36]	1492	1481 [36]	0.15 ± 0.09	n.d.	0.6 ± 0.31	n.d.
17	trans, trans-α-Farnesene ^b^	1746	1744 [36]	1505	1504 [36]	n.d.	n.d.	4 ± 1.4	8 ± 2.5
18	Methyl salicylate ^b^	1772	1768 [36]	1198	1193 [36]	n.d.	n.d.	0.3 ± 0.15	1.6 ± 0.70
19	trans-Nerolidol ^a^	2038	2036 [36]	1564	1561 [36]	n.d.	n.d.	1.4 ± 0.30	10 ± 1.1
20	Methyl anthranilate ^b^	2241	2255 [40]	–	–	n.d.	n.d.	0.3 ± 0.10	3 ± 1.5
21	Indole ^a^	2446	2440 [36]	1300	1298 [36]	0.4 ± 0.31	n.d.	2.2 ± 0.64	18 ± 4.4

^a^ Confirmatory identification with reference material. ^b^ Tentative identification based on the comparison of experimental linear retention indices (LRI) with those from the literature and of mass spectra with those of NIST and Wiley databases (EI, 70 eV, matching > 90%). n.d., not detected.

## Data Availability

Supporting data are included in the CIBIMOL research group data repository.

## Supplementary Materials

The following are available online, Figure S1: Mass spectra (EI, 70 eV) of **A**. The compound with LRI 1381 (DBWAX) detected in the volatiles emitted by *C. nitens* leaves after herbivory. **B**. Isobutyraldoxime reference compound (Sigma-Aldrich, Cat. #S388378, CAS 151-00-8). **C**. Isobutyraldoxime from NIST, 2017 database. Figure S2: Mass spectra (EI, 70 eV) of **A**. The compound with LRI 1479 (DB-WAX) detected in the volatiles of *C. nitens* leaves after insect attack. **B**. 2-Methylbutyraldoxime (NIST, 2017). **C**. 3-Methylbutyraldoxime (NIST, 2017). Differences between isomeric aldoximes based on the McLafferty rearrangement typical products.
