# Supplementary material for: Volatiles Emission by Crotalaria nitens after Insect Attack
Source: Molecules. 2021 Nov 17;26(22):6941. doi: 10.3390/molecules26226941 (PMC8618423; doi:10.3390/molecules26226941)
Supplement: Supplementary file 1 [file molecules-26-06941-s001.zip › molecules-1401918-SI.pdf]

Supplementary Materials

Volatiles Emission by *Crotalaria nitens* after Insect Attack

Fausto Prada [1,2], Elena E. Stashenko [1,2,*] and Jairo René Martínez [1,2]

[1] Center for Chromatography and Mass Spectrometry (CROM-MASS), Universidad Industrial de Santander, 680002 Bucaramanga, Colombia; fausto.prada@correo.uis.edu.co (F.P.); jmartine@uis.edu.co (J.R.M.)
[2] Colombia Research Center for Biomolecules (CIBIMOL), Universidad Industrial de Santander, 680002 Bucaramanga, Colombia
* Correspondence: elena@tucan.uis.edu.co

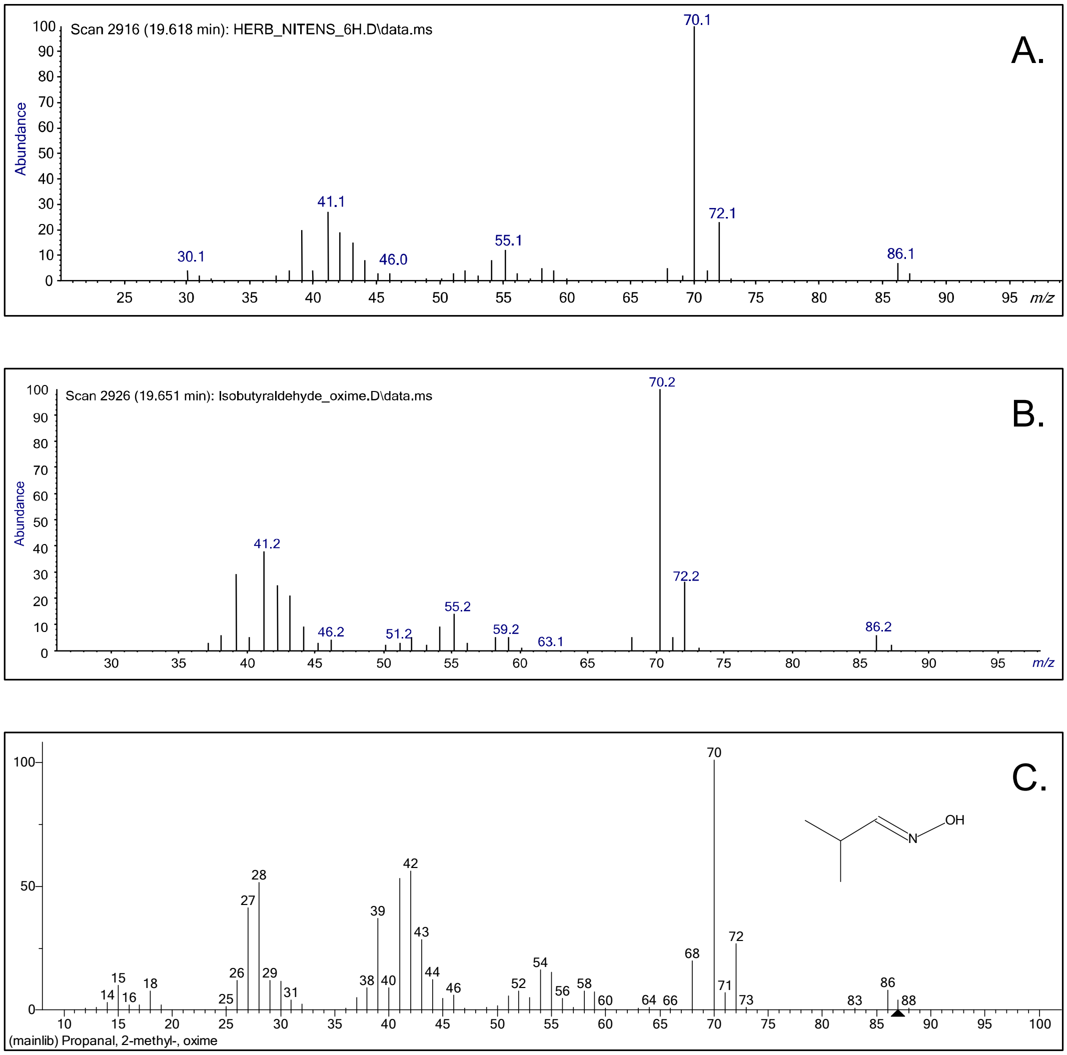

Figure S1. Mass spectra (EI, 70 eV) of (**A**) The compound with LRI 1381 (DBWAX) detected in the volatiles emitted by *C.nitens* leaves after herbivory. (**B**) Isobutyraldoxime reference compound (Sigma-Aldrich, Cat. #S388378, CAS 151-00-8).(**C**). Isobutyraldoxime from NIST, 2017 database.

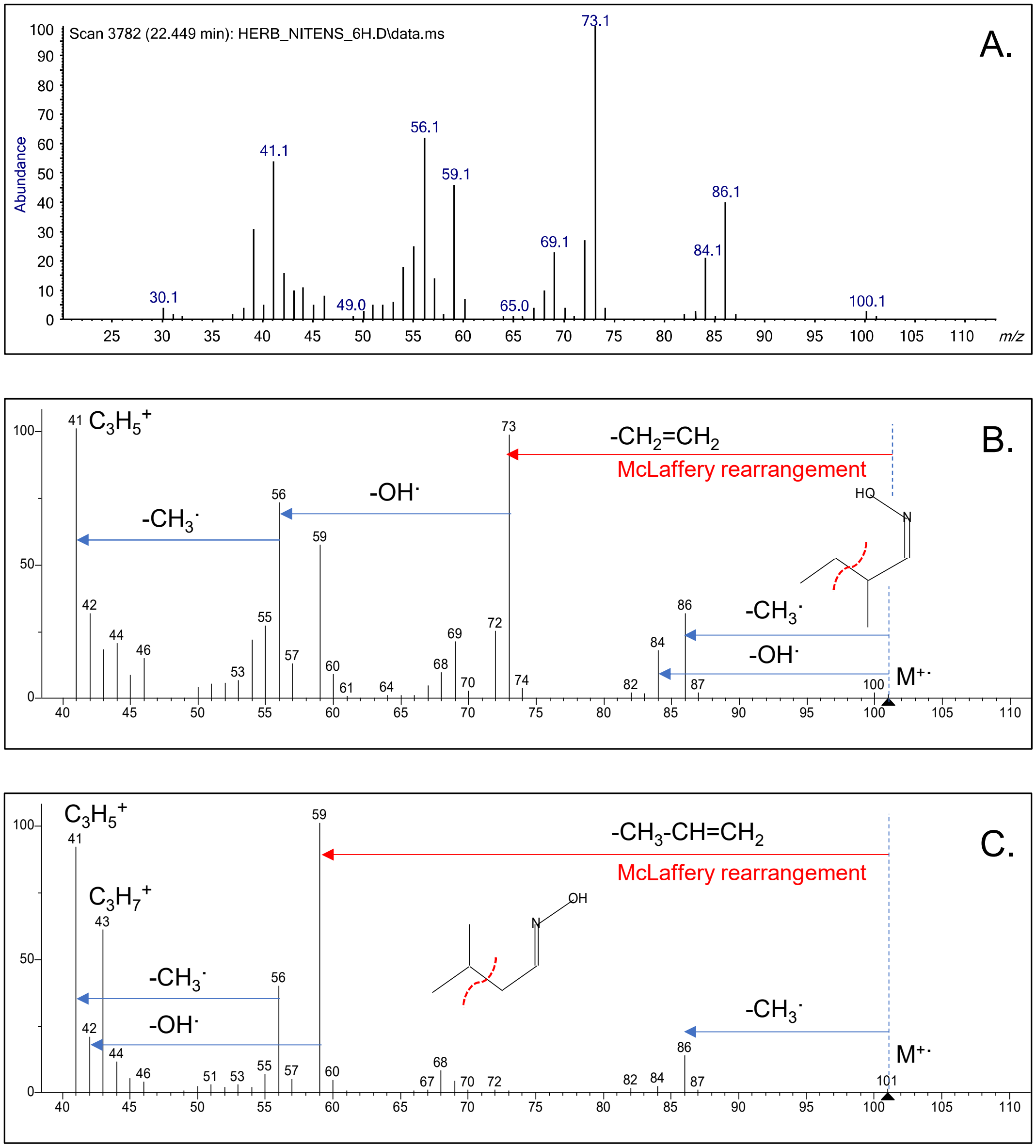

Figure S2. Mass spectra (EI, 70 eV) of (**A**) The compound with LRI 1479 (DB-WAX) detected in the volatiles of *C. nitens* leaves after insect attack. (**B**) 2-Methylbutyraldoxime (NIST, 2017). (**C**) 3-Methylbutyraldoxime (NIST, 2017). Differences between isomeric aldoximes based on the McLafferty rearrangement typical products.
